# Gastric GIST with life-threatening anaemia and paraganglioma: a rare presentation of suspected Carney–Stratakis syndrome in a young adult

**DOI:** 10.1093/jscr/rjag517

**Published:** 2026-06-26

**Authors:** Nakul Ganju, Chukwunonso Ndulue, Heera Tesfaye Erega, Kristin Slater, Sarrah Fadul, Juan Carlos Santiago-Gonzalez, Farshad Aduli, Babak Shokrani

**Affiliations:** Department of Surgery Gastroenterology, Howard University Hospital, 2041 Georgia Ave NW, Washington, DC 20060, United States; Department of Surgery Gastroenterology, Howard University Hospital, 2041 Georgia Ave NW, Washington, DC 20060, United States; Department of Surgery Gastroenterology, Howard University Hospital, 2041 Georgia Ave NW, Washington, DC 20060, United States; Department of Surgery Gastroenterology, Howard University Hospital, 2041 Georgia Ave NW, Washington, DC 20060, United States; Department of Surgery Gastroenterology, Howard University Hospital, 2041 Georgia Ave NW, Washington, DC 20060, United States; Department of Surgery Gastroenterology, Howard University Hospital, 2041 Georgia Ave NW, Washington, DC 20060, United States; Department of Surgery Gastroenterology, Howard University Hospital, 2041 Georgia Ave NW, Washington, DC 20060, United States; Department of Surgery Gastroenterology, Howard University Hospital, 2041 Georgia Ave NW, Washington, DC 20060, United States

**Keywords:** GIST, Carney–Stratakis syndrome, paraganglioma, SDH-deficient, iron deficiency anaemia, subtotal gastrectomy

## Abstract

Gastrointestinal stromal tumours (GISTs) rarely occur in young adults; in this age group, succinate dehydrogenase (SDH)-deficient subtypes predominate and may signal hereditary tumor syndromes. We present a 23-year-old male with a prior carotid paraganglioma who developed life-threatening iron deficiency anaemia (haemoglobin 4.0 g/dl) as the presenting manifestation of a high-grade gastric GIST. Subtotal gastrectomy with Billroth II reconstruction achieved R0 resection. Molecular testing was negative for KIT and PDGFRA mutations. Given the patient’s age, mixed epithelioid/spindle histology, wild-type molecular profile, and paraganglioma history, Carney–Stratakis syndrome was suspected. SDHB immunohistochemistry confirmed an SDH-deficient phenotype, and germline testing identified a pathogenic heterozygous SDHx variant, establishing the diagnosis of Carney–Stratakis syndrome. At follow-up, the patient was clinically stable and enrolled in a multidisciplinary surveillance program. This case highlights the importance of recognizing atypical hematologic presentations of GIST and the need for comprehensive molecular and genetic evaluation in young patients.

## Introduction

Gastrointestinal stromal tumours (GISTs) are the most common mesenchymal neoplasms of the gastrointestinal tract, arising from interstitial cells of Cajal [1]. While most arise in older adults and harbor activating KIT or PDGFRA mutations, ~10%–15% are wild-type [2]. Among these, succinate dehydrogenase (SDH)-deficient GISTs disproportionately affect young patients and may herald hereditary syndromes such as Carney-Stratakis syndrome (CSS)—defined by the co-occurrence of GIST and paraganglioma due to germline SDH subunit mutations [3, 4]. Life-threatening anaemia as the sentinel presentation is uncommon and can delay recognition. We describe the surgical management and post-operative surveillance strategy for a young patient with suspected CSS presenting in this atypical fashion.

## Case report

A 23-year-old male with a history of left carotid paraganglioma (Shamblin type II) resected in 2023 presented with a 2-week history of chest pain, exertional dyspnea, palpitations, and an episode of hematemesis. Haemoglobin was 4.0 g/dl with serum iron <10 μg/dl and ferritin 0.8 ng/ml, consistent with profound iron deficiency anaemia. Computed tomography (CT) abdomen/pelvis demonstrated a 3.9 × 4.2 × 5.8 cm lobulated, heterogeneously enhancing mass along the lesser curvature with an exophytic component and no peritoneal metastases.

**Figure 1:**
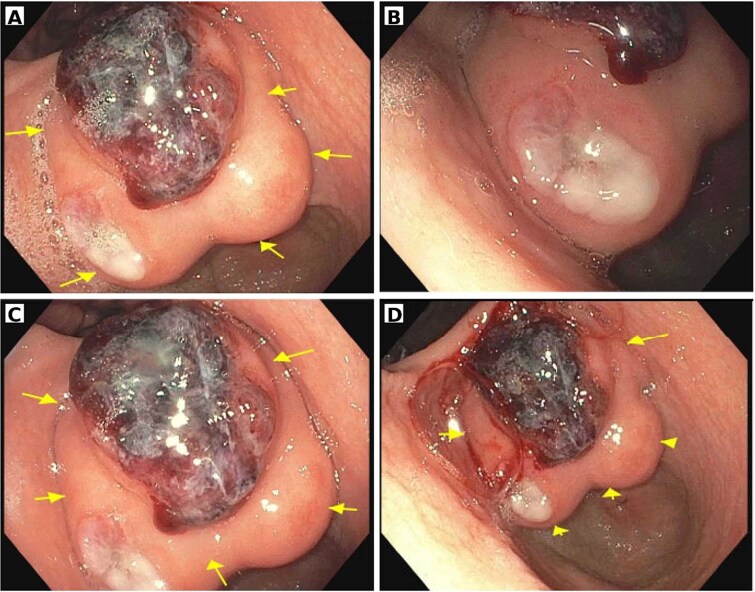
Esophagogastroduodenoscopy demonstrating a large gastric antral mass with lobulated, ulcerated, and necrotic surface (A, C; yellow arrows demarcate margins), submucosal endoluminal component (B), and active mucosal bleeding (D, arrow) consistent with hematemesis.

Following transfusion of three packed red blood cell units, urgent esophagogastroduodenoscopy identified a malignant-appearing gastric antral mass with irregular, ulcerated mucosa (Fig. 1). Biopsy demonstrated chronic active gastritis with ulceration.

The patient underwent exploratory laparotomy with subtotal gastrectomy, Billroth II reconstruction, Braun anastomosis, jejunostomy tube placement, and omental flap coverage. Intraoperative findings confirmed a large exophytic mass along the lesser curvature without peritoneal or hepatic involvement. Post-operatively, he tolerated diet advancement and maintained haemoglobin 9.5–10.5 g/dl.

**Figure 2:**
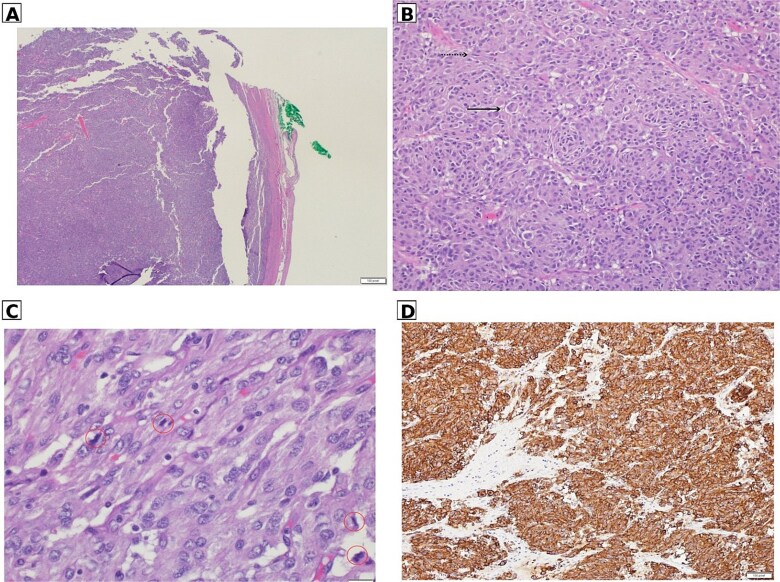
Histopathology of resected gastric GIST. (A) Low-power H&E: Submucosal mass with intact serosa. (B) Medium-power H&E: Mixed epithelioid (solid arrow) and spindle cell (dotted arrow) morphology. (C) High-power H&E: Brisk mitotic activity (circled), 12 mitoses/5 mm^2^. (D) CD117 immunostaining: Diffuse cytoplasmic positivity.

**Figure 3:**
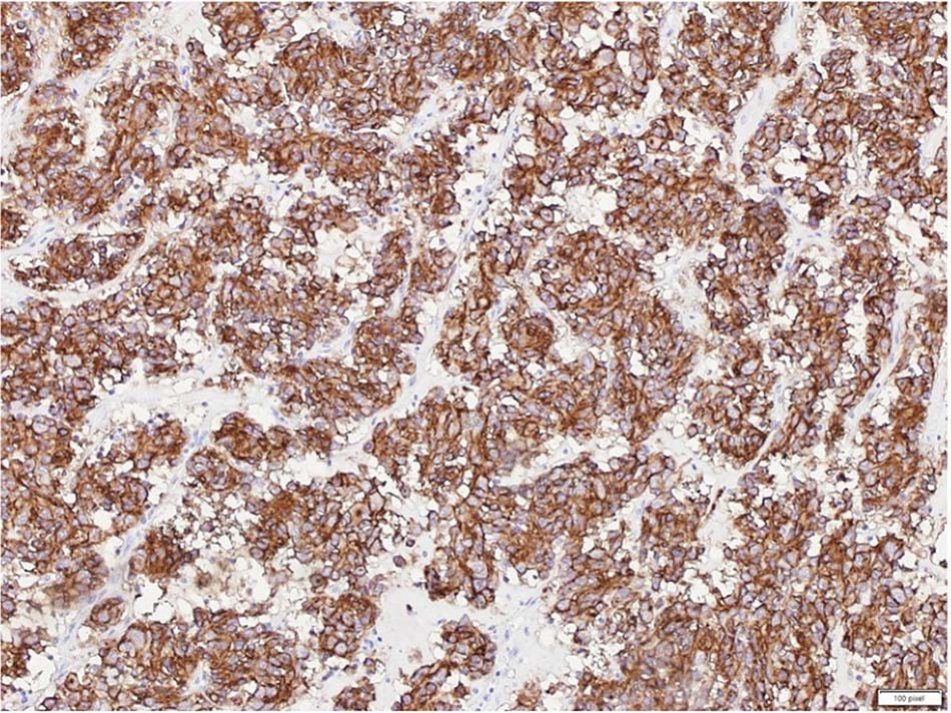
DOG1 immunohistochemistry showing diffuse membranous and cytoplasmic positivity, confirming GIST diagnosis.

Final pathology revealed a 6.5 × 4.6 × 3.7 cm GIST, mixed epithelioid and spindle cell type, high grade (G2), with 12 mitoses per 5 mm^2^ (Fig. 2). All margins and three lymph nodes were negative (pT3N0). CD117, CD34, and DOG1 were positive (Fig. 3). Next-generation sequencing returned negative for KIT (exons 2, 3, 8–11, 13, 14, 17–19) and PDGFRA (exons 5, 6, 11, 12, 14, 16, 18). *H. pylori* immunostaining on background mucosa was positive. Given the patient’s age, wild-type molecular profile, mixed morphology, and paraganglioma history, SDH-deficient GIST with CSS was suspected. SDHB immunohistochemistry demonstrated loss of cytoplasmic staining in tumor cells with preserved internal control staining, confirming an SDH-deficient phenotype. Germline testing identified a pathogenic heterozygous SDHx variant, establishing the diagnosis of Carney-Stratakis syndrome. At follow-up, the patient remained clinically stable with improving postoperative anaemia, tolerance of oral intake, and no clinical evidence of recurrent gastrointestinal bleeding. He was enrolled in a multidisciplinary surveillance program including serial CT or magnetic resonance imaging (MRI) of the abdomen and pelvis every 3–6 months, whole-body functional imaging to evaluate for occult paraganglioma or pheochromocytoma, and annual biochemical testing with plasma or urine metanephrines/catecholamines.

## Discussion

This case highlights three instructive features. First, life-threatening anaemia (haemoglobin 4.0 g/dl) as the primary presentation of gastric GIST is unusual. Profound unexplained iron deficiency anaemia in young patients should prompt aggressive endoscopic and cross-sectional imaging evaluation, even when classic GI symptoms are absent.

Second, the wild-type KIT/PDGFRA molecular profile, mixed epithelioid histology, young age, and concurrent paraganglioma history are collectively highly suggestive of SDH-deficient GIST and CSS [4, 5]. CSS is autosomal dominant, caused by germline SDH subunit mutations (SDHB/C/D), and is characterized by the dyad of GIST and paraganglioma [4, 6]. SDH-deficient GISTs respond poorly to imatinib, making accurate molecular subtyping critical for systemic therapy decisions [3, 7].

Third, the confirmed SDH-deficient phenotype and pathogenic germline SDHx variant establish this as CSS with direct management implications. A structured multidisciplinary surveillance plan was implemented: serial CT or MRI of the abdomen and pelvis every 3–6 months during the initial postoperative period given high-risk pathology (pT3, G2, 12 mitoses/5 mm^2^), then annually thereafter [8]. Whole-body ^68^Ga-DOTATATE PET/CT has been scheduled to assess for occult paraganglioma or pheochromocytoma [9]. Annual 24-hour urine catecholamines and metanephrines are being monitored given the paraganglioma history. Once germline results are available, first-degree relatives will be referred for genetic counseling and SDH mutation testing [6]. At follow-up, the patient remained clinically stable with improving postoperative anaemia, tolerance of oral intake, and no clinical evidence of recurrent gastrointestinal bleeding.

Surgical resection with R0 margins remains the cornerstone of localized GIST management [10]. This case underscores that in young patient with wild-type GIST and a personal or family history of paraganglioma, CSS should be suspected, SDH IHC and germline testing pursued, and a multidisciplinary surveillance framework established at the time of surgery.
